# The X-Rays in Dentistry—A Whimsey

**Published:** 1896-06

**Authors:** Frank W. Sage


### The X Rays in Dentistry—A Whimsey.

BY FRANK W. SAGE, D.D.S.

Abstract of a paper read before the Mississippi Valley Dental Society, April, 1896.

In November of last year, in the town of Wurzburg, Germany, was made a discovery which seems destined to confer upon the science of surgery a power of benefiting the race in even a greater degree than has the comparatively recent discovery of Lister.

It is a fact significant, and worthy of note, that the first suggestions as to the value of this important discovery had reference to its availability in surgery. Aside from casual suggestions as to the use of the rays in the arts—their application to the testing of castings, welding metals, *et id genus omne*—attention seems, by common consent, to have turned to their use as an invaluable adjunct to surgery. This is natural and befitting, since the addition to our knowledge of so invaluable an aid to the prevention or alleviation of human suffering, must be conceded to outweigh any consideration of possible advantage which might accrue to the sciences and arts on the side of mere utility and convenience. Far more important is the consideration that the surgeon may be aided in a manner hitherto beyond the dream of possibility, in examining tumors, all affected tissues of internal organs, *e. g.*, malformations, fractures, abscesses, impacted or encysted foreign bodies, etc. Far more important is this than that the artisan should be enabled to detect hidden flaws in metals, or that the photographer should find at his command a new experiment to excite the dilettante's curiosity by a feat of magic surpassing the most wonderful of any school of mystics.

But in all the many lucubrations on the nature and the possible availability of the mysterious X rays, not a word has the author of this screed seen in any publication, magazine or newspaper, touching the benefits likely to accrue to the dentist or to the dental patient. The dental journals of recent date contain one or two articles on the great discovery, but singularly enough not a hint of promised value to our profession is to be found in them. And yet, the merest casual reflection, it would seem, should have suggested the probability that this discovery is destined to work a revolution in the matter of diagnosis alone, infinitely more important than any late discovery in pathology, histology or therapeutics. Now, that Mr. Edison promises us shortly a perfected instrument for revealing—not by photography, but by the immediate and direct application of the X rays to the patient's hand, foot or jaw—a clear view of hidden lesions, what a clearing away of the cobwebs of mystery shall we witness! What a relegating to the garret of volumes on diagnosis! What an overhauling of theories and speculations on etiology and prognosis! What a change in forthcoming literature on all branches of scientific dental research! Nor is this mere idle speculation. From various quarters come tidings of successful experiments forestalling Mr. Edison in the line of his special inquiry, so that it seems not extravagant to expect before long a simple electrical appliance to be attached to the dentist's outfit far more indispensable than the electric motor, the electric mouth lamp, the cautery, the mallet or the baking furnace.

How shall we restrain our impatience to get to work with the new appliance? Behold the pyorrhœa alveolaris enthusiast dropping his pen, heedless of splashing ink, and sending post-haste for the patient whom he has burnt oftenest with zinc chloride, argentum nitras, acid sulph., and with nervous haste turning on the X rays to note the exact extent of the reparative process, or locating calcic nodules, pus pockets, galleries, etc. Fancy him reaching after a calcic spicula at the very apex of a root, applying the delicate scaler to the exact spot, undisturbed by any accidental flow of blood or pus, through which the rays continue to shed their light, as before. Behold him locating the bit of protruding wire or broken-off broach, in a root, noting the curve of roots requiring extraction, observing the deposition of granular matter in the abscess from which he has withdrawn his tampon, locating sequestra, detecting exostoses, pulp nodules, necroses, antral engorgements or odontomes. With what facility will he fit crowns to roots, peer into approximal spaces for suspected cavities, examine cervical borders of fillings. Away with carbon paper, and such clumsy aids in securing proper fits of crowns to roots, of crowns to crowns. Farewell to the recording ledger. What need of noting on paper that this root was filled with gold, that one with tin, or the other with chloro-percha? The X rays will tell us all about it, nay, more invaluable even than this will be their service—they will tell us what the patient's former dentist, on the next street, in the next town, in the unknown beyond, has done.

In the October, 1886, number of the Cincinnati *Medical and Dental Journal*, the writer of this paper published a whimsical article foreshowing the probable status of dentistry at the end of the twentieth century. An agent is represented as calling upon a dentist offering for sale various patented appliances, one of which he describes in the language here quoted:

"Doctor, this little instrument is called the 'Pocket-Book and Bank Account Detective.' Attached to your chair, it records on this little dial, in characters intelligible to yourself alone, the exact amount of cash in your patient's pocket. By the use of this simple device you are enabled to preserve a conscience void of offense, since your fee need never exceed the patient's ability to pay," etc., etc.

Little did the writer suspect when he wrote the above the announcement, which would in less than ten years be made, that the contents of a purse, concealed in a pocket, could be distinctly photographed.

We can conceive of the consternation of one tempted to suggest the value of the X rays to dentists when he pictures himself seated in the operator's chair, his thoughts concentrated on that "buzzing machine," or that inquisitorial clamp, while the dentist's assistant stealthily turns the X rays on his artfully concealed purse, in order to supply data upon which the busy operator may, later on, assign a fee for the service commensurate with the victim's means. But then, on the other hand, we can conceive of the patient himself armed with a Kodak X Ray apparatus, stealthily peering into the operator's brain with a view to penetrate the centers of thought exposing to view the unrighteous purpose of charging ten dollars for a one dollar filling.

But from the standpoint of mutual advantage to dentist and patient what a glorious future is about to open to the profession. The practical use of the X rays is destined to overcome the patient's reluctance to submit to the dentist's assistant in the matter of diagnosticating obscure lesions, since he must perceive that the merest tyro employing the X rays, has an advantage over most experienced diagnostician in ferreting out the cause of trouble.

"Doctor, I have wandering pains about my face and jaw, can't say whether it is neuralgia or toothache," says the suffering patient coming in upon the busy operator.

"Ah, that's not very pleasant," says the operator over his shoulder, without looking round. "John, turn the X rays upon Mrs. Brown's jaw, and report to me what you discover." In a few minutes John reports: "Upper third of left lower second molar pulp decomposed under filling. No periostitis as yet."

"Ah, very good, you may treat the case, John."

Which John accordingly does, to Mrs. Brown's perfect satisfaction. No waste of time whatever. The operator goes tranquilly on with his case in hand leaving Mrs. Brown in John's charge, thinking of the former days when he was interrupted every fifteen minutes during the day by some Mrs. Brown, who wouldn't trust John on any account, and who kept him ten minutes testing the suspected tooth with ice-water, hot gutta-percha, percussion, etc., no one, in the end, being any the wiser. Here comes Mrs. Jackson complaining that her new store teeth are a misfit. Turn on the rays; hand Mrs. Jackson a mirror, and let her point out, if she can, any point at which the plate fails of being in close conjunction with the underlying tissues. Equally applicable are the X rays in detecting faults of articulation.

Welcome the dawn of the glorious day when the new agent, by revealing the perfection of the dentist's work, shall force upon the consciousness of the complainant that an unpaid bill is the only real lingering element of dissatisfaction.

Thus hastily and crudely have we sketched a few of the benefits which the X rays may confer upon our profession. And who shall say that a literal fulfillment is beyond reasonable expectation?

